# *Lactobacillus murinus* Alleviates High Fructose‐Induced MASLD by Boosting Arginine Production

**DOI:** 10.1002/fsn3.71502

**Published:** 2026-02-17

**Authors:** Xinglin Mo, Guilin Zhao, Lanlan Liu, Lan Zhen, Qing Huang, Yue Wang, Xiaopan Yang, Linfei Huang, Luming Wan, Congwen Wei, Ruzhou Zhao, Jie Hu, Yong Li, Jing Yuan, Chenke Ma, Feixiang Wu

**Affiliations:** ^1^ Hepatobiliary Surgery Department Guangxi Medical University Cancer Hospital Nanning Guangxi China; ^2^ Laboratory of Advanced Biotechnology Beijing Institute of Biotechnology Beijing China; ^3^ Key Laboratory of Early Prevention and Treatment for Regional High Frequency Tumor Guangxi Medical University Nanning Guangxi China; ^4^ Department of Clinical Laboratory, The Third Medical Centre Chinese PLA General Hospital Beijing China; ^5^ Department of Critical Care Medicine, The First Medical Centre Chinese PLA General Hospital Beijing China; ^6^ National‐Local Joint Engineering Research Center of Biodiagnostic & Biotherapy, The Second Affiliated Hospital Xi'an Jiaotong University Xi'an China; ^7^ Frontier Anti‐Aging and Cell Therapy Research Institute Xi'an China; ^8^ Capital Institute of Pediatrics Beijing China; ^9^ Medical Supplies Center Chinese PLA General Hospital Beijing China

**Keywords:** arginine, fructose, *Lactobacillus murinus*, MASLD, metabolites

## Abstract

Over the past few years, the prevalence of high‐fructose diets has become a significant inducer of metabolic dysfunction‐associated steatotic liver disease (MASLD). The effects and pathological mechanisms of high dietary fructose on gut microbiota and its subsequent role in MASLD remain unclear. Here, we investigated the impact of fructose supplementation on MASLD progression in wild‐type C57BL/6 mice using both high‐fructose drinking water and a high‐fructose diet. Through 16S rDNA sequencing, we observed that long‐term fructose exposure significantly reduced the abundance of *Lactobacillus murinus* ( *L. murinus* ) in the intestines of mice. We aim to further elucidate the role and underlying mechanisms of *L. murinus* in high‐fructose‐induced MASLD. Logically, we supplemented *L. murinus* exogenously in the high‐fructose mouse model and found that *L. murinus* significantly alleviated fructose‐induced MASLD symptoms, characterized by reduced liver ballooning, inflammation, hepatic cholesterol, triglycerides, as well as decreased blood cholesterol, triglycerides, ALT and AST levels. To further investigate the mechanistic basis of *L. murinus* ‐mediated protection, we conducted serum and fecal metabolomic analyses. These studies identified arginine as the sole metabolite exhibiting marked reductions in both serum and intestinal compartments. Integrated multi‐omics analysis revealed a strong positive correlation between gut *L. murinus* abundance and arginine levels. ELISA demonstrated that exogenous administration of *L. murinus* effectively restored circulating arginine concentrations in high‐fructose‐fed mice. Importantly, direct arginine supplementation produced similar therapeutic benefits as *L. murinus* . Specifically, it improved key features of MASLD, including reduced liver ballooning, less liver inflammation, lower buildup of cholesterol and triglycerides in the liver and blood, and decreased ALT/AST levels. These data revealed a novel mechanism underlying fructose‐induced MASLD by decreasing the abundance of gut *L. murinus* , which disrupts arginine metabolism. *L. murinus* and arginine could serve as potential therapeutic strategies against fructose‐induced MASLD.

## Graphical abstract

Long‐term high‐fructose diet significantly reduced the abundance of *Lactobacillus murinus* in the intestines of mice. *Lactobacillus murinus* was found to be significantly associated with arginine metabolism. Exogenous supplementation of *Lactobacillus murinus* alleviated fructose‐induced MASLD and restored serum arginine levels, and direct arginine supplementation exerted comparable therapeutic effects.

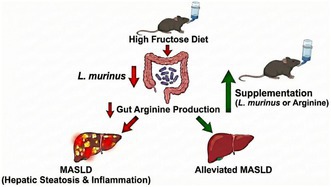

## 1 Introduction

In recent years, with the prevalence of processed foods and sugary beverages, the intake of fructose has significantly increased (Tappy and Lê 2010; Herman and Birnbaum 2021; Noubiap et al. 2022). Fructose, a monosaccharide widely found in high‐fructose corn syrup, candies, soft drinks, and various processed foods, has been linked to various health issues such as insulin resistance, obesity, and chronic inflammation with prolonged high intake (Hannou et al. 2018). Fructose is primarily metabolized in the liver and small intestine (Jang et al. 2018; Geidl‐Flueck et al. 2021). Unlike glucose, which is phosphorylated by glucokinase (GK), fructose is phosphorylated by ketohexokinase (KHK) and further metabolized at a faster rate (Helsley et al. 2020). Research indicates that fructose catabolism occurs up to 10 times faster than glucose catabolism and is not regulated by its end products or insulin (Asipu et al. 2003; Helsley et al. 2020). In fructose catabolism, the intermediate products glyceraldehyde (GA) and dihydroxyacetone phosphate (DHAP) serve dual functions. On one hand, they integrate into the glucose metabolic pathway as shared intermediates, thereby facilitating glucose catabolism. On the other hand, they can be converted into key intermediates such as glycerol, glycerate, and pyruvate, which are crucial substrates for de novo lipogenesis (DNL) in the liver, contributing to lipid synthesis from scratch (Jensen et al. 2018; Helsley et al. 2020; Geidl‐Flueck and Gerber 2023). As a result, fructose is more likely than glucose to contribute to the development of metabolic disorders such as obesity and metabolic dysfunction‐associated steatotic liver disease (MASLD) (Stanhope et al. 2009; Softic et al. 2017; Jang et al. 2018). A deeper exploration of the underlying mechanisms by which fructose induces MASLD could provide potential intervention strategies for the prevention and treatment of MASLD, holding substantial clinical significance.

The gut microbe‐mediated “gut‐liver axis” plays a crucial role in regulating the progression of MASLD (Leung et al. 2016; Tilg et al. 2022). Dysbiosis of gut microbiota can lead to abnormalities in hepatic lipid metabolism, thereby promoting the development of MASLD. Studies have found that patients with MASLD typically have lower gut microbiota diversity, and the abundance of certain specific bacterial groups may be associated with the occurrence and severity of MASLD (Fukui 2019; Arab et al. 2020). It has been reported that short‐chain fatty acids (SCFAs), metabolites of gut microbiota, can significantly influence liver metabolic functions (Jang et al. 2018). Gut microbiota, including *Lactobacillus*, *Bifidobacterium*, and *Saccharomyces boulardii*, have been reported to ameliorate symptoms of obesity and MASLD (Cui et al. 2022; Si et al. 2022; Song, Zhang, et al. 2023; Wen et al. 2023; Lau et al. 2024). Among these, *Lactobacillus*, a genus that includes both probiotic and non‐probiotic species, has been extensively studied for its therapeutic potential. Notably, well‐characterized probiotic strains such as *L. rhamnosus* , *L. acidophilus* , and *L. plantarum* have been shown to exhibit beneficial effects in the context of MASLD (Si et al. 2022; Wen et al. 2023; Lau et al. 2024). *L. rhamnosus* improves metabolic control mainly by modulating the gut–glucagon–insulin axis (Han et al. 2022), whereas *L. rhamnosus* ameliorates dyslipidaemia to a statin‐like extent without targeting hepatic lipogenesis directly (Zafar et al. 2022). The flagship strain *L. rhamnosus* persists in the intestinal tract and enhances systemic immunity via cGAS/STING‐type‐I‐interferon signaling, but reports no consistent reduction in liver fat accumulation (Mahalak et al. 2022; Si et al. 2022). *L. acidophilus* suppresses NAFLD‐associated hepatocellular carcinoma by secreting valeric acid that inhibits NF‐κB‐mediated inflammation (Lau et al. 2024), while *L. plantarum* mitigates high‐fat‐diet‐induced steatosis through butyrate‐activated AMPK (Zhu et al. 2024). These strains thus act through immunomodulation, lipid‐lowering or short‐chain fatty acid signaling. Modulating the gut microbiota homeostasis and supplying probiotics have emerged as new strategies for preventing and treating MASLD. These strategies highlight the close association between gut health and liver function, providing an important perspective for exploring the pathogenesis of MASLD.

Notably, *L. murinus* has emerged as a key mediator in gut‐liver axis communication, demonstrated to ameliorate systemic inflammation and enhance intestinal barrier integrity (Pan et al. 2018). A recent study further revealed that intestinal *L. murinus* is essential for the protective effects of time‐restricted feeding against septic liver injury (Hu et al. 2025). It orchestrates this protection by driving the production of the metabolite 3‐hydroxybutyrate (3‐HB), which subsequently activates the PI3K/AKT/mTOR/LPIN1 signaling pathway to suppress hepatocyte ferroptosis. Beyond its role in liver protection and gut barrier, *L. murinus* also exhibits significant immunomodulatory capabilities, as shown by its ability to activate lung Vγ4+ γδ T cells and enhance IL‐17A‐mediated resistance against bacterial infections (Zhang et al. 2025). Therefore, we hypothesized that a high‐fructose diet might disrupt a specific *L. murinus* ‐mediated pathway, contributing to MASLD development.

L‐arginine (L‐arg) is a multifunctional amino acid and a major intestinal metabolite in mammals and microbiota, playing a crucial role in host–microbe interactions (Nüse et al. 2023). Within mammals, L‐arg serves as a precursor in various metabolic pathways, regulating cell division and growth. Research has revealed close associations between arginine metabolism and glucose and lipid metabolism, thereby exerting regulatory effects on the progression of diabetes and obesity (Hu et al. 2017). Besides, in a colitis rodent model, fructose induces gut dysbiosis and disrupts arginine metabolism (Song, Gan, et al. 2023). Whether gut microbiota‐derived arginine affects fructose‐induced MASLD progression remains unclear.

Based on a long‐term high‐fructose diet‐induced MASLD mouse model, this study identified a novel strain, *L. murinus* , that significantly reduced post high‐fructose diet via 16 s microbiome analysis. After analysis of the metabolomic profiles of colonic contents and serum, we found that arginine was significantly decreased in both feces and serum. Microbiome‐metabolome co‐analysis revealed a significant correlation between *L. murinus* and arginine. Further experiments confirmed that exogenous supplementation of *L. murinus* markedly increased serum arginine levels and significantly alleviated symptoms of high fructose‐induced MASLD. In conclusion, this study reveals that *L. murinus* ‐involved arginine metabolism plays a crucial role in the progression of high fructose‐induced MASLD. *L. murinus* or arginine may serve as potential probiotics or supplements to mitigate high fructose‐induced MASLD.

In summary, the widespread consumption of high‐fructose diets has been closely linked to the rising prevalence of MASLD, yet the specific roles of gut microbiota and their functional metabolites in this process remain incompletely understood. This study aims to investigate whether specific gut microbial species and their associated metabolic alterations contribute critically to the progression of high‐fructose‐induced MASLD. We hypothesized that a high‐fructose diet induces specific disruptions in the gut microbiome, and that one or more resulting metabolic deficiencies might play a causal role in liver pathology. To test this, we employed an integrated multi‐omics approach combined with targeted bacterial supplementation in a mouse model. We specifically focused on identifying which bacterial species were consistently depleted by fructose and which metabolites were consequently altered, thereby uncovering potential mechanistic links within the gut‐liver axis. Elucidating such a pathway would not only provide novel insights into the pathogenesis of fructose‐induced MASLD but could also reveal specific microbial or metabolic targets for future therapeutic interventions.

## 2 Materials and Methods

### 2.1 Animal Model

7‐week‐old male C57BL/6J mice (specific‐pathogen free SPF grade, weighted 21‐23 g) were purchased from Spearfish (Beijing) Biotechnology Co. Ltd. All mice were housed in a Specific Pathogen‐Free (SPF) environment, maintained at a temperature of 25°C, with 60% relative humidity, and a 12‐h light–dark cycle.

For chow diet (CD) group, mice were given standard laboratory chow diet (BiotechHD, HD000) and water. In the high‐fructose drinking water (HFrD) group, mice were given access to drinking water containing 30% fructose (Macklin, D809612) for 2 months. In the high‐fructose diet group, mice were fed a diet containing 20% fructose (BiotechHD, HD061a) for 2 months. For *L. murinus* intervention group (HFrD+ *L. murinus* ), mice were supplied with drinking water containing 30% fructose and subjected to daily intragastric gavage of a *L. murinus* (ATCC, 35020) solution (1 × 10^9^ CFU/d in 100 μL PBS) over a two‐month period. For arginine intervention group (HFrD+Arg.), mice were provided with drinking water containing 30% fructose for 2 months and were administered arginine (Sigma, A5006) at a dose of 2 mg/g body weight daily during the last 30 days. Mice treated with 30% fructose water for 2 months served as the control for mice in the HFrD+ *L. murinus* group and the HFrD+Arg. group.

### 2.2 Sample Collection

At the experimental endpoint, mice were anesthetized by intraperitoneal administration of 1% sodium pentobarbital. Blood samples were collected through orbital venous plexus puncture using sterile microcentrifuge tubes without anticoagulant and allowed to clot at room temperature for 30 min. Following centrifugation at 3000*g* for 15 min at 4°C, the supernatant serum fraction was carefully collected for subsequent analyses. Liver tissues were immediately excised and processed for histopathological examination and biochemical assays. Simultaneously, freshly feces samples were collected in sterile microcentrifuge tubes, flash‐frozen in liquid nitrogen, and maintained at.

−80°C until 16S rDNA sequencing analysis.

### 2.3 Histological Analysis

Formalin‐fixed liver tissue was fixed in 10% neutral buffered formalin (Sigma‐Aldrich, HT501128) and routinely processed through graded ethanol dehydration, xylene clearing, and paraffin embedding. Sections were stained with H&E staining kit (Solarbio, G1120) for morphological assessment. The images were captured with a 3 DHISTECH Digital slice scanning system (Pannoramic, 3DHISTECH Ltd., Hungary). NASH activity score (NAS), including steatosis, intralobular inflammation, and hepatocellular ballooning, were blindly evaluated by the histological scoring system as previously described (Kleiner et al. 2005).

Steatosis grade was assessed based on the percentage of hepatocytes containing lipid droplets. Hepatocellular ballooning was evaluated by quantifying enlarged hepatocytes with rarefied cytoplasm. The NAFLD Activity Score (NAS) was calculated as the sum of these three components (steatosis+inflammation+ballooning) following the established histological criteria (Kleiner et al. 2005).

The fresh cleaned Liver tissues were embedded in OCT compound (Biosharp, BL557A) within appropriate embedding molds, with sufficient embedding medium added to cover the tissue with a 2–3 mm layer. The embedded tissues were rapidly frozen in liquid nitrogen. After complete solidification of the OCT medium, the frozen blocks were removed from the molds and mounted on specimen chucks using additional OCT compound for fixation. The mounted samples were returned to the cryostat for final freezing. All necessary instruments including fine brushes, microtome blades, and anti‐roll plates were pre‐cooled in the cryostat chamber prior to sectioning. Sectioning was performed using a cryostat (Leica CM1950, Shanghai Leica Instrument Co. Ltd.) with the chamber temperature maintained between −15°C and −20°C. Initial coarse trimming was conducted at 50–100 μm thickness until the tissue surface was properly exposed. Subsequently, sections were cut at the experimentally required thickness and carefully collected on glass slides (Jiangsu Shitai Experimental Equipment Co. Ltd., 188105). The collected sections were air‐dried at room temperature for 30 min before being stored at −80°C for subsequent experimental use.

For lipid visualization, frozen liver sections were stained with Oil Red O kit (G1262, Solarbio) following the manufacturers' protocol. The Oil Red O‐positive area was quantified using ImageJ software (1.47v, NIH, USA) with threshold‐based color segmentation as previously described (Schneider et al. 2012), and expressed as a percentage of total tissue area.

For histological analysis, three liver sections from different anatomical regions of each mouse were evaluated as technical replicates, with the mean value calculated per animal. Each experimental group included 6 mice serving as biological replicates for subsequent statistical analysis.

### 2.4 Biochemical Index Detection

Serum biochemical parameters including aspartate aminotransferase (AST) (Beijing Ruilda Biotechnology Co. Ltd., 220105), alanine aminotransferase (ALT) (Beijing Ruilda Biotechnology Co. Ltd., 210902), total cholesterol (TC) (Beijing Ruilda Biotechnology Co. Ltd., 231213), and triglycerides (TG) (Beijing Ruilda Biotechnology Co. Ltd., 211015) were quantified using commercial enzymatic assay kits (Beijing Ruilda Biotechnology Co. Ltd., China) according to the manufacturer's protocols. All measurements were performed in duplicate using an L100 semi‐automatic biochemical analyzer (Shanghai Kehua Bio‐engineering Co. Ltd., China) with appropriate quality controls.

Serum arginine content was measured by Arginine ELISA Assay Kit (Abcam, ab241028) according to the manufacturer's protocols.

Total cholesterol (Solarbio, BC1985) and triglycerides (Solarbio, BC0625) were extracted from liver tissue homogenates using chloroform‐methanol (2:1, v/v) and measured using colorimetric ELISA kits following the manufacturer's instructions. Absorbance was read at 500 nm using a microplate reader (BioTek Synergy H1, USA).

### 2.5 Gut Microbiota Analysis

Fresh fecal samples were collected from mice in each group. Bacterial genomic DNA was extracted from these samples using the QIAamp Power Fecal Pro DNA Kit (QIAGEN, Germany), following the manufacturer's instructions. The V3–V4 regions of the microbial 16S rRNA genes were subsequently amplified using specific primers (forward: 5′‐CCTACGGGNGGCWGCAG‐3′; reverse: 5′‐GGACTACHVGGGTATCTAAT‐3′). The amplification protocol included an initial denaturation step at 95°C for 5 min, followed by 30 cycles of denaturation at 72°C for 1 min, and a final extension at 72°C for 7 min. Amplicons were quantified and pooled in equal concentrations for sequencing on the Illumina MiSeq platform (Illumina Inc., CA, USA). Gene Denovo (Guangzhou, China) performed DNA extraction, quality assessment, library construction, and high‐throughput sequencing, and collaborated with us on the processing and analysis of the 16S rRNA sequencing data. In summary, FASTP (version 0.18) was utilized to filter the clean reads, specifically removing those with an average Phred score below 20, those containing adapter sequences, and those with more than 10% unknown nucleotides (N). Subsequently, Operational Taxonomic Units (OTUs) were clustered at a similarity threshold exceeding 97% using UPARSE (version 9.2.64). The resulting OTU sequences were annotated for taxonomic classification utilizing the SILVA database (version 138.1). Furthermore, the OmicShare tools (https://www.omicshare.com/tools/) provided by Gene Denovo were employed to conduct analyses of gut microbiota composition.

### 2.6 Metabolomic Analysis of Colonic Contents

Colonic contents were homogenized with extraction solvent (methanol: acetonitrile: water, 2:2:1, v/v/v) via 30 s vortex mixing. Magnetic beads were added to the mixture, followed by 10 min grinding at 45 Hz and 10 min sonication in an ice‐water bath. The homogenate was incubated at −20°C for 1 h, then centrifuged at 12,000 rpm (4°C, 15 min). The supernatant was lyophilized using a vacuum concentrator. Dried metabolites were reconstituted in acetonitrile: water (1:1, v/v), vortexed for 30 s, sonicated for 10 min (ice‐water bath), and centrifuged under identical conditions. A 10 μL aliquot of each sample was injected for instrumental analysis.

Analysis was performed using a UHPLC (Vanquish UHPLC, Thermo) coupled to an Orbitrap Exploris 480 in Gene Denovo Biotechnology Co. Ltd. (Guangzhou, China). For HILIC separation, samples were analyzed using a 2.1 mm × 100 mm ACQUIY UPLC BEHAmide 1.7 μm column (waters, Ireland). To minimize the potential influence of instrumental signal drift, all samples were analyzed in randomized order. Quality control (QC) samples, prepared by pooling equal aliquots of all study samples, were interspersed throughout the analytical sequence to monitor system stability and ensure data reliability. Raw data were converted to .mzML format using ProteoWizard, followed by peak alignment, retention time correction, and peak area extraction using the XCMS program. The following XCMS parameters were applied: For peak picking: centWave *m*/*z* = 25 ppm, peakwidth = *c* (10, 60), prefilter = *c* (10, 100); For peak grouping: bw = 5, mzwid = 0.025, minfrac = 0.5. Subsequently, metabolite identification and data preprocessing were conducted, including missing value filtering (removing ions with > 50% missing values), missing value imputation (KNN method), and feature filtering (removing features with RSD > 50%).

Multivariate analysis was performed in SIMCA‐*P*+ 14.1 (Umetrics, Umeå, Sweden). Orthogonal projections to latent structures‐discriminant analysis (OPLS‐DA) models were validated through 7‐fold cross‐validation, with model quality assessed using *R* ^2^ *Y* (goodness‐of‐fit) and *Q* ^2^ (predictive ability) parameters. Metabolites with variable importance in projection (VIP) scores > 1.0 and *p* < 0.05 (Student's *t*‐test) were considered statistically significant. Pathway enrichment analysis was conducted using the KEGG database (https://www.kegg.jp/kegg/pathway.html), with false discovery rate (FDR) correction for multiple testing.

### 2.7 Serum Metabolomics Analysis

After mixing 100 μL of serum with pre‐cooled 80% methanol, the supernatant was incubated on ice for 5 min and centrifuged at 15,000*g* for 20 min at 4°C. The supernatant was then incubated for 5 min at 4°C and centrifuged at 15,000*g* for 20 min. The resulting supernatant was diluted to a final concentration of 53% methanol using LC–MS grade water. The sample was then transferred to a new centrifuge tube and centrifuged again at 15,000*g* for 20 min at 4°C. Finally, UHPLC–MS/MS analysis was performed as before.

### 2.8 Statistical Analysis

Statistical analysis and graph generation were performed using GraphPad Prism 9.0 (GraphPad Software, San Diego, CA, USA). Serum liver function indices, liver biochemical indices, and the area of liver tissue positive for Oil Red O staining were analyzed using an unpaired two‐tailed Student's *t*‐test. Results are expressed as mean ± standard error of the mean (SEM) unless otherwise specified. Statistical significance was defined as *p* < 0.05.

### 2.9 Fecal DNA Was Extracted and Subjected to 16S rRNA qPCR

Genomic DNA was extracted from fecal samples using the QIAamp DNA Stool Mini Kit (Qiagen) following the manufacturer's protocol. To improve bacterial community representation, a mutanolysin (Sigma‐Aldrich) digestion step was incorporated by adding 6 μL of 25 kU mL^−1^ stock solution per sample. Quantitative PCR was performed on a 7500 Sequence Detector (Applied Biosystems) to determine bacterial 16S rRNA gene copy numbers in the extracted DNA. The 10 μL reaction mixtures contained 1× SYBR Green Master Mix (Applied Biosystems), 300 nM of each primer, and 4 ng of genomic DNA. Total bacterial 16S rRNA gene levels were quantified using universal primers Univ 337F (5′‐ACTCCTACGGGAGGCAGCAGT‐3′) and Univ 518R (5′‐GTATTACCGCGGCTGCTGGCAC‐3′). For specific detection of *Lactobacillus murinus* , primers LactoMF (5′‐TCGAACGAAACTTCTTTATCACC‐3′) and LactoMR (5′‐CGTTCGCCACTCAACTCTTT‐3′) were employed. All measurements were conducted in duplicate.

## 3 Results

### 3.1 High‐Fructose Induced Hepatic Steatosis and Impaired Liver Function

**FIGURE 1:**
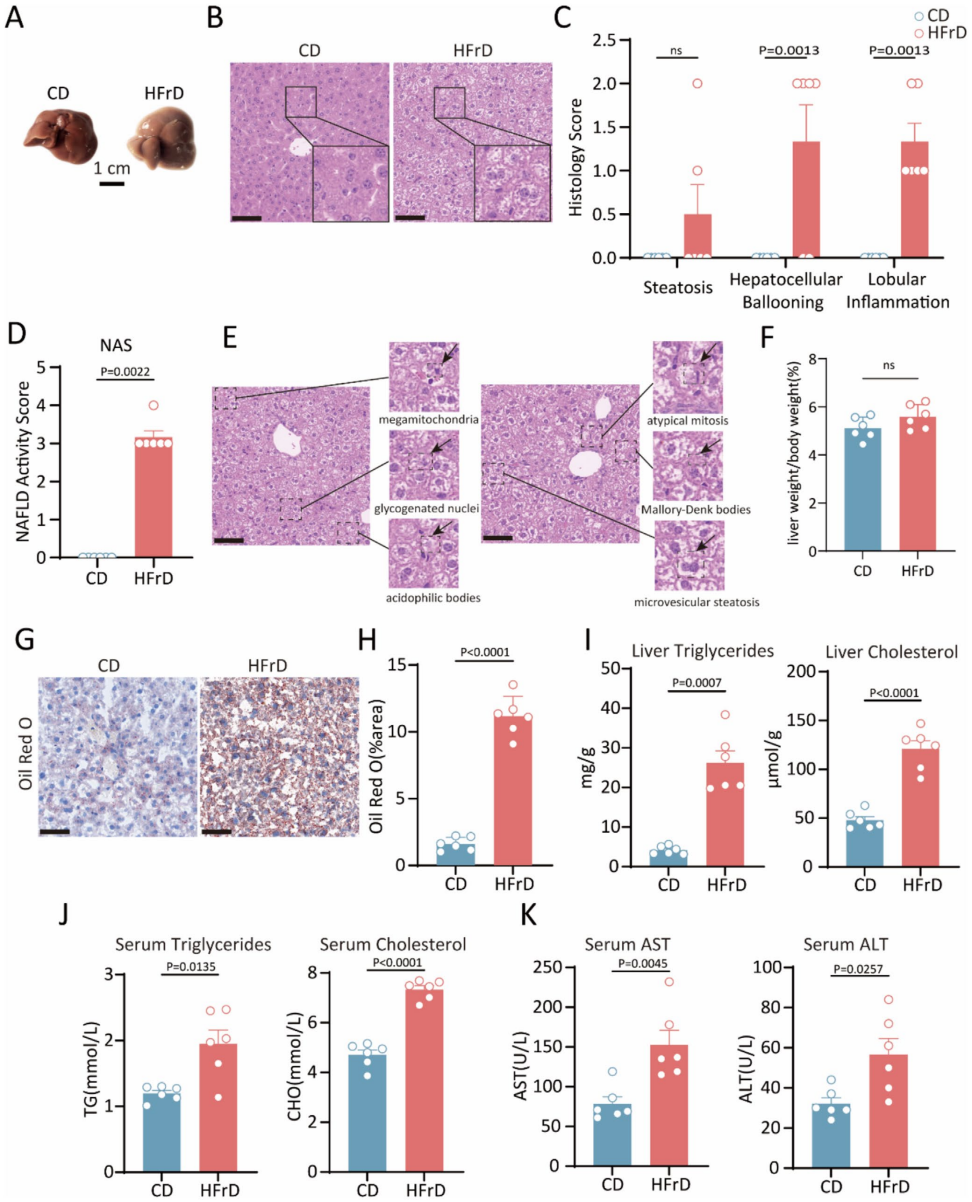
High‐fructose induced hepatic steatosis and liver injury in mice. (A) Representative pictures for livers from CD and HFrD mice. Scale bar, 1 cm. (B) H&E staining of liver sections. Scale bar, 60 μm. (C) Individual histological scores for hepatic steatosis, hepatocellular ballooning, and lobular inflammation. (D) NAFLD activity scores (NAS). (E) Representative images of histological features of hepatic steatosis in liver H&E sections from HFrD mice. Scale bar, 60 μm.(F) The liver/body weight ratio. (G, H) Representative images of liver Oil Red O staining and quantitative analysis of lipid droplets area using Image J. Scale bar, 60 μm. (I, J) Assessment of liver and serum triglycerides and cholesterol levels using ELISA. (K) Biochemical analysis of serum ALT and AST. Data are mean ± SEM, compared to CD mice, using unpaired *t*‐test. Data were collected from 6 mice per group for (A–K).

Long‐term consumption of a high‐fructose diet has been reported to cause liver damage and increased accumulation of hepatic lipids (Softic et al. 2017; Jensen et al. 2018). To determine this phenomenon, we developed a mouse model of high‐fructose drinking (HFrD). After 12 weeks of HFrD, HFrD mice developed pronounced mega and yellowish liver (Figure 1A). However, the liver‐to‐body weight ratio showed no statistically significant difference between the CD and HFrD groups (Figure 1F), suggesting that the initial liver hypertrophy might not be fully reflected by absolute organ weight normalization under these dietary conditions. H&E staining demonstrated marked steatohepatitis in HFrD mice (Figure 1B), characterized by elevated histological scores for hepatocellular ballooning and lobular inflammation. Despite no statistically significant difference in hepatic steatosis, this pathological progression resulted in a significant increase in NAS scores (Figure 1C,D). Particularly, other significant morphological features of hepatic steatosis were observed in the livers of HFrD mice, including megamitochondria, glycogenated nuclei, acidophilic bodies, atypical mitosis, Mallory‐Denk bodies, and microvesicular steatosis (Figure 1E). Moreover, Oil Red O staining further confirmed a significant increase in liver lipid accumulation in HFrD mice (Figure 1G). Then the content of triglycerides and cholesterol in serum and liver tissues were determined. The results showed that both hepatic and serum triglyceride and cholesterol levels were significantly higher in the HFrD mice than in the CD mice (Figure 1I,J). Biochemical analysis revealed that fructose‐treated mice exhibited notably elevated serum levels of alanine transaminase (ALT) and aspartate transaminase (AST), markers of hepatic function, compared to the control group (Figure 1K). Together, the results presented above indicate that high‐fructose diet induced hepatic fat accumulation and impaired liver function.

### 3.2 Effects of Long‐Term High‐Fructose Diet or High‐Fructose Drinking on Gut Microbiome Profile in C57 Mice

**FIGURE 2:**
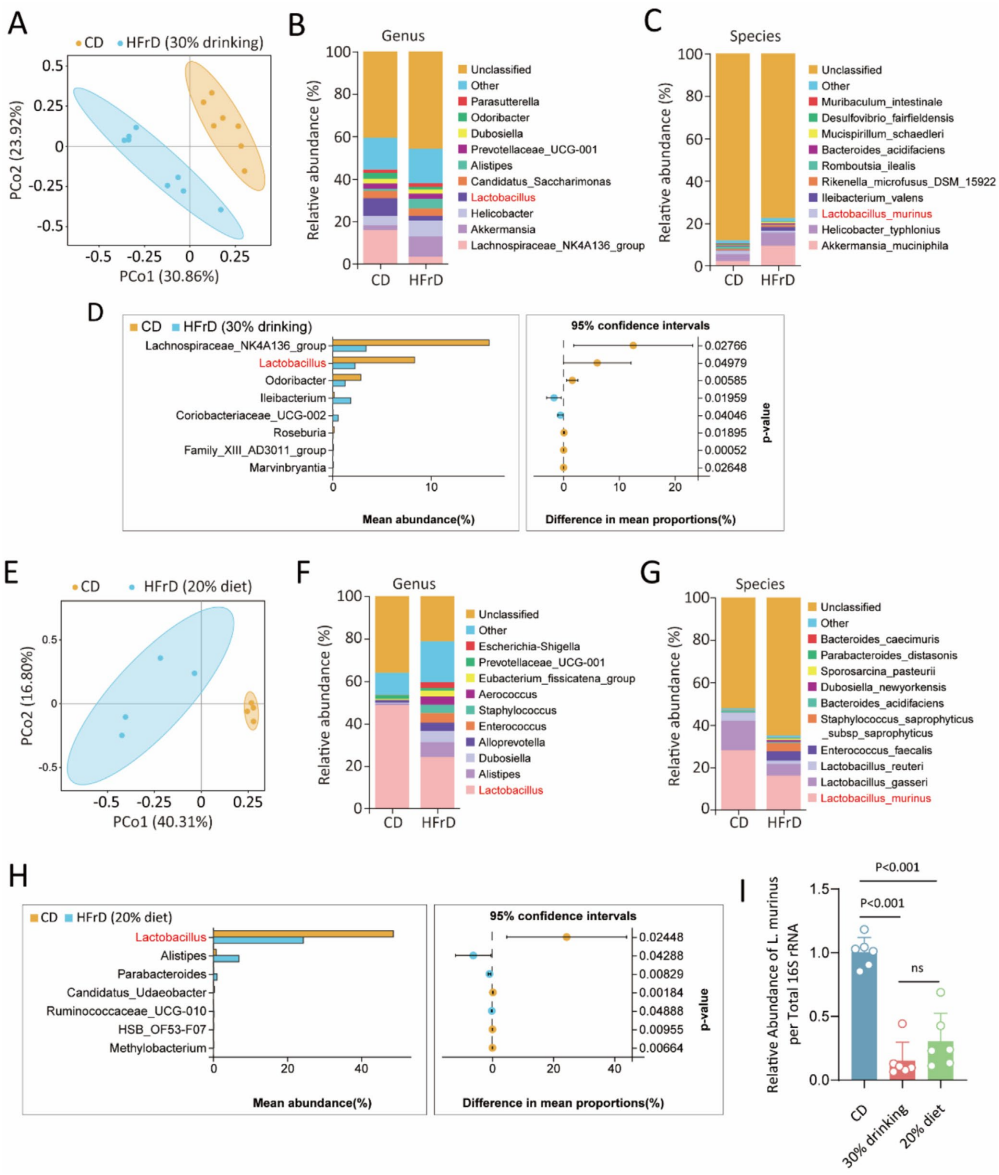
Long‐term high‐fructose diet or high‐fructose water altered the gut microbiome profile, especially decreased the abundance of *L. murinus* . (A) The PCoA score plot of colonic microbiota between 30% fructose drinking group and corresponding control group based on the bray index. (B, C) The stacked bar graph of gut microbiota abundance at the genus and species level. (D) Statistical analysis of indicated microbiota at the genus level by Welch's *t*‐test. (E–H) Comparative analysis of the gut microbiome between the 20% fructose diet group and the corresponding control group using PCoA analysis (E), species stacking analysis at genus (F) and species (G) level, and statistical *t*‐test of indicated microbiota at the genus level (H). (I) qPCR detected the relative abundance of *L. murinus* in feces under two fructose feeding regimens. All results were compared to CD group. *n* = 7–8 mice per group for (A–D). *n* = 4 mice per group for (E–H). *n* = 6 mice per group for (I).

Dysbiosis of gut microbiota plays a significant role in the development of diet‐induced hepatic steatosis (Leung et al. 2016; Fukui 2019; Sun et al. 2019). Thus, we employed 16S rDNA gene sequencing to investigate the impact of high‐fructose drinking on the abundance and diversity of intestinal microbiota. PCoA analysis was conducted to examine the composition alteration of gut microbiota based on the data. The results showed a significantly distinct clustering distribution between HFrD group and CD group, suggesting that HFrD may alter the composition and diversity of gut flora at the OTU level (Figure 2A). To further clarify the specific alterations in gut microbiota abundance, the genus and species level of taxonomic distribution were studied. As shown in genus level, *Alistipes*, *Lactobacillus*, *Akkermansia*, and *Lachnospiraceae_NK4A136* were the dominant gut flora in both control and fructose‐drinking mice (Figure 2B). Statistical *t*‐test results verified that *Lachnospiraceae_NK4A136*, *Lactobacillus*, and *Odoribacter* were notably decreased in fructose‐drinking mice (Figure 2D). The relative abundance of *Ileibacterium_valens*, *L. murinus* , and *Akkermansia_muciniphila* was significantly changed between the two groups (Figure 2C). To confirm the microbiota alteration caused by high fructose diet, we employed a high‐fructose dietary mice model and conducted 16S rDNA sequencing. The PCoA plot showed clearly distinct clustering between the high‐fructose diet group and the control group (Figure 2E). The stacked graph and *t*‐test analyzed at genus level displayed that *Lactobacillus* was primarily altered bacteria, characterized by decreased *L. murinus* , *L. gasseri* , and *L. reuteri* (Figure 2F–H).

A direct comparison of the gut microbiota alterations induced by high‐fructose drinking and a high‐fructose diet revealed both model‐specific and conserved responses. For instance, Lachnospiraceae_NK4A136, Lactobacillus, and Odoribacter were notably reduced only in the HFrD‐drink model (Figure 2D), while the HFrD‐diet model led to a more pronounced and isolated depletion within the Lactobacillus genus (Figure 2H). Despite these model‐specific variations in the secondary microbial signatures, the most significant and consistent finding was the severe and shared impairment of the Lactobacillus genus. As shown in the overall community structures (Figure 2B,F), Lactobacillus was the only genus that was markedly affected in both models. This convergence of evidence identifies Lactobacillus depletion as the central, protocol‐independent microbial consequence of high‐fructose exposure.

To specifically and quantitatively validate the reduction of *L. murinus* , we performed qPCR analysis on intestinal contents from CD, 30% high‐fructose water, and 20% high‐fructose diet groups. The results demonstrated a significant decrease in *L. murinus* abundance in fructose‐fed groups compared to the CD group, with no statistically significant difference between the two fructose feeding regimens (Figure 2I). Together, these findings demonstrate that decreased abundance of Lactobacillus, particularly *L. murinus* , represents a key alteration in gut microbiome profiles induced by both high‐fructose drinking and high‐fructose diet. The consistent reduction of *L. murinus* across different fructose exposure models, confirmed by species‐specific qPCR analysis, highlights its specific susceptibility to fructose‐induced microbial dysbiosis.

### 3.3 *L. murinus* Administration Ameliorated Hepatic Steatosis and Restored Liver Function in High Fructose‐Induced MASLD Mice

**FIGURE 3:**
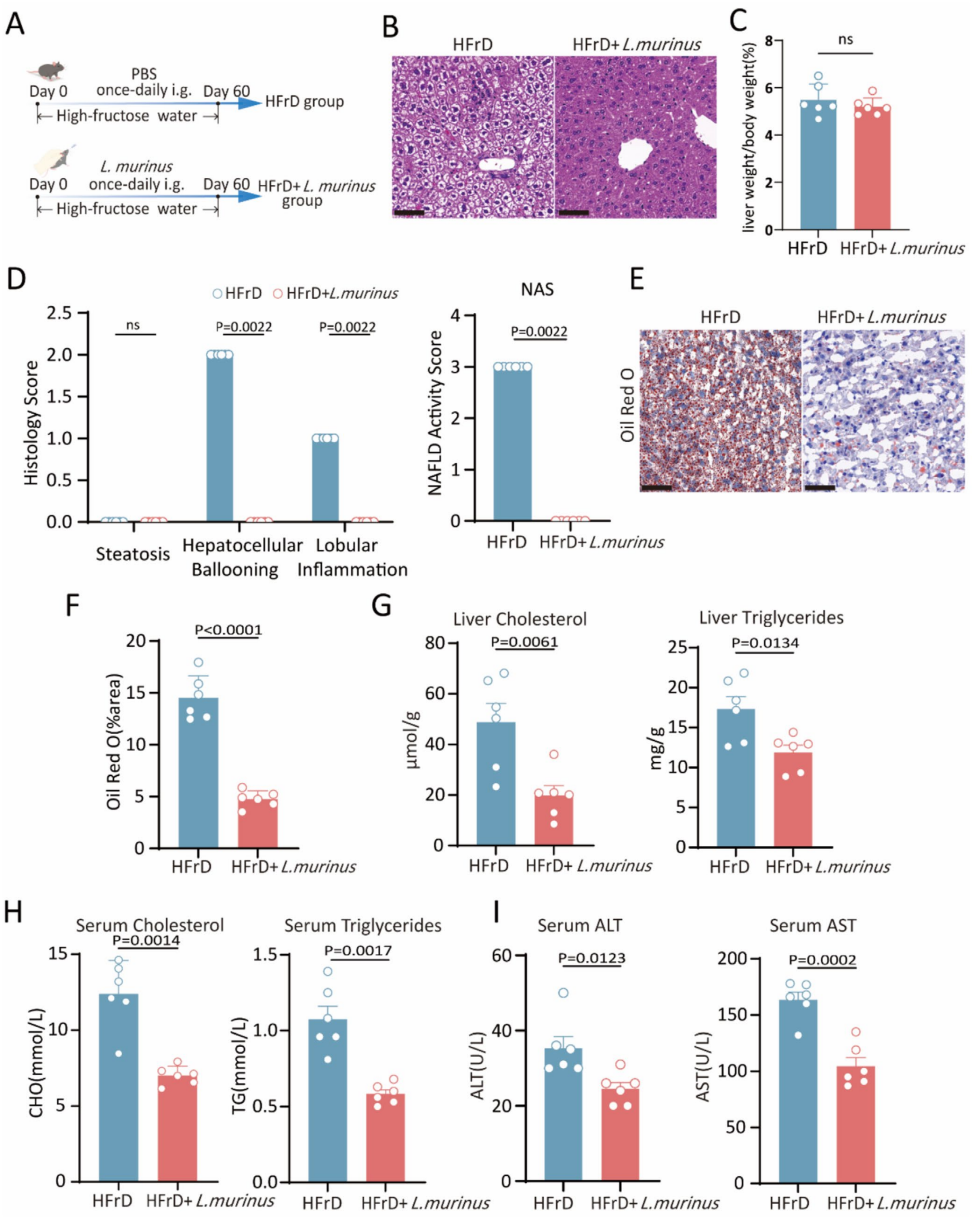
*L. murinus* administration ameliorated hepatic steatosis and restored liver function in high fructose‐induced NAFLD mice. (A) Schematic representation of mice supplemented with *L. murinus* . (B) H&E staining of liver tissues. Scale bar, 60 μm. (C) The liver/body weight ratio. (D) Histological scores of NAFLD activity‐related indicators. (E, F) Representative images of Oil Red O staining in liver sections (E) and quantitative analysis of lipid droplet area (F). Scale bar, 60 μm. (G) ELISA assay for liver triglycerides and cholesterol. (H) Biochemical assay for serum triglycerides and cholesterol. (I) Biochemical assay for serum ALT and AST. Data represent mean ± SEM, compared to HFrD control mice, using an unpaired *t*‐test. Data were collected from 6 mice per group for (A–I).

To elucidate the relationship between *L. murinus* and fructose‐induced MASLD, we constructed a high‐fructose induced MASLD model supplemented with *L. murinus* (Figure 3A). H&E staining results showed that HFrD mice supplemented with *L. murinus* for 8 weeks exhibited notable reduced MASLD activity, indicated by decreased histological scoring of hepatocellular ballooning and lobular inflammation, compared to the HFrD control mice (Figure 3B,D). The liver‐to‐body weight ratio showed no statistically significant difference between the HFrD group and the HFrD group supplemented with *L. murinus* (Figure 3D), The liver of mice supplemented with *L. murinus* exhibited a significant decrease in lipid droplets, confirmed by Oil Red O staining (Figure 3E,F). Furthermore, the levels of triglycerides and cholesterol in serum and liver tissues were markedly reduced in HFrD mice treated with *L. murinus* (Figure 3F,H). The hepatic function was restored in *L. murinus* ‐treated mice, characterized by lower serum ALT and AST compared to HFrD control mice (Figure 3I).

### 3.4 High Fructose Disrupted Host–Microbe Arginine Metabolism Through the Altered *L. murinus*

**FIGURE 4:**
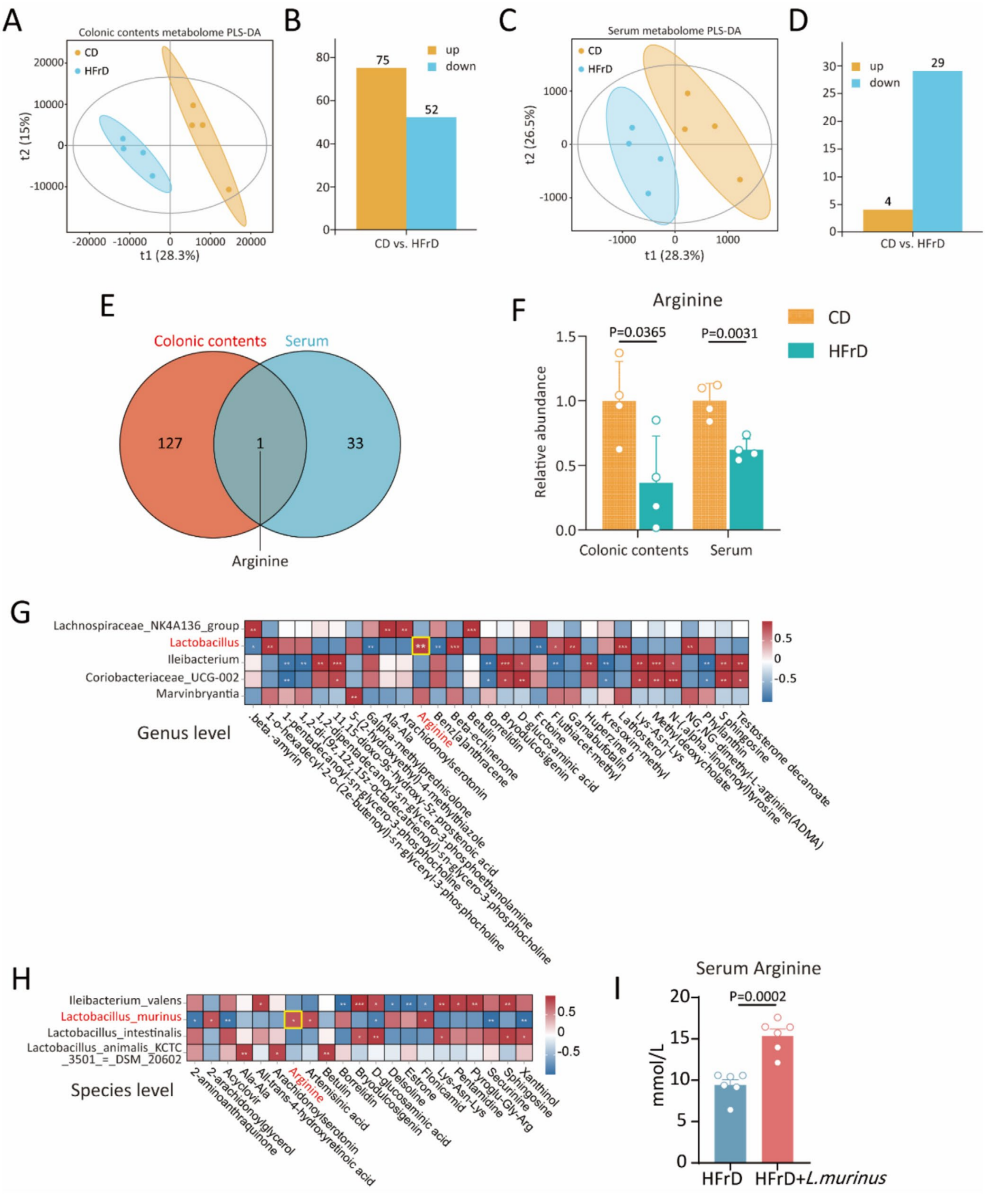
High fructose disrupted host–microbe arginine metabolism through the altered *L. murinus* . (A) PLS‐DA plot of colonic contents in CD and HFrD group. (B) Bar graph of significantly different metabolites in colonic contents. (C) PLS‐DA score plot of serum in different groups. (D) Bar graph of significantly different metabolites in serum. (E) Venn diagram of differential metabolites in colonic contents and serum. (F) The relative abundance of arginine in colonic contents and serum. (G and H) Analysis of Pearson's correlation coefficient between altered bacteria at the different levels and metabolites. (I) ELISA assay for serum arginine. Data represent mean ± SEM compared to corresponding control, using unpaired *t*‐test. Screening criterion in (C and D), *p* < 0.05, fold change ≥ 1.5. *n* = 4 mice per group for metabolomics. *n* = 6 mice in each group for (I).

The metabolic activity of the gut microbiota exerts significant effects on host metabolism through host–microbe interactions (Adak and Khan 2018; Fan and Pedersen 2020). To evaluate the metabolic profile changes affected by high‐fructose, we performed metabolomic technology to screen metabolites in colonic contents and serum. PLS‐DA was initially used to investigate the overall metabolic variations among samples. According to PLS‐DA plots, a distinct clustering in HFrD group was observed in both colonic contents and serum, compared to CD control group (Figure 4A,C). A total of 127 significantly altered (*p* < 0.05, fold change > 1.5) metabolites were identified after comparing the colonic contents from the CD control group and the HFrD group, including 75 upregulated and 52 downregulated metabolites (Figure 4B). In terms of the serum metabolomics, 33 metabolites with significant changes (*p* < 0.05, fold change > 1.5) between the two groups were identified, comprising 4 upregulated metabolites and 29 downregulated metabolites (Figure 4D).

To identify the potential relationship between gut microbiome metabolism and serum metabolism, we explored the metabolites that were concurrently altered in both colonic contents and serum based on the significantly altered metabolites. Strikingly, only one metabolite, arginine, was identified, showing a significant decrease in relative abundance in both colonic contents and serum of the HFrD group compared to the CD control group (Figure 4E,F). Arginine has been reported to be a pivotal interface of mutual host–microbe metabolism interactions (Nüse et al. 2023). To investigate the connection between arginine and the altered Lactobacillus, a joint analysis of the gut microbiome and metabolome was performed. We employed Pearson's correlation to analyze the relationships between significantly changed gut microbiota and metabolites. Our analysis revealed a significant positive correlation between arginine levels and *Lactobacillus* abundance within the genus‐level microbiota that exhibited significant alterations (Figure 4G). Moreover, *L. murinus* was the only one positively correlated with arginine at the species level (Figure 4H). The ELISA experiment showed that serum arginine levels were restored after supplementation with *L. murinus* in HFrD mice, further confirming the relationship of *L. murinus* and arginine (Figure 4I). Together, these data revealed that fructose‐induced dysregulation of host arginine metabolism was closely associated with the decreased abundance of gut *L. murinus* caused by long‐term high‐fructose intake.

### 3.5 L‐Arginine Exhibited Protective Function Against MASLD in High Fructose Mice

**FIGURE 5:**
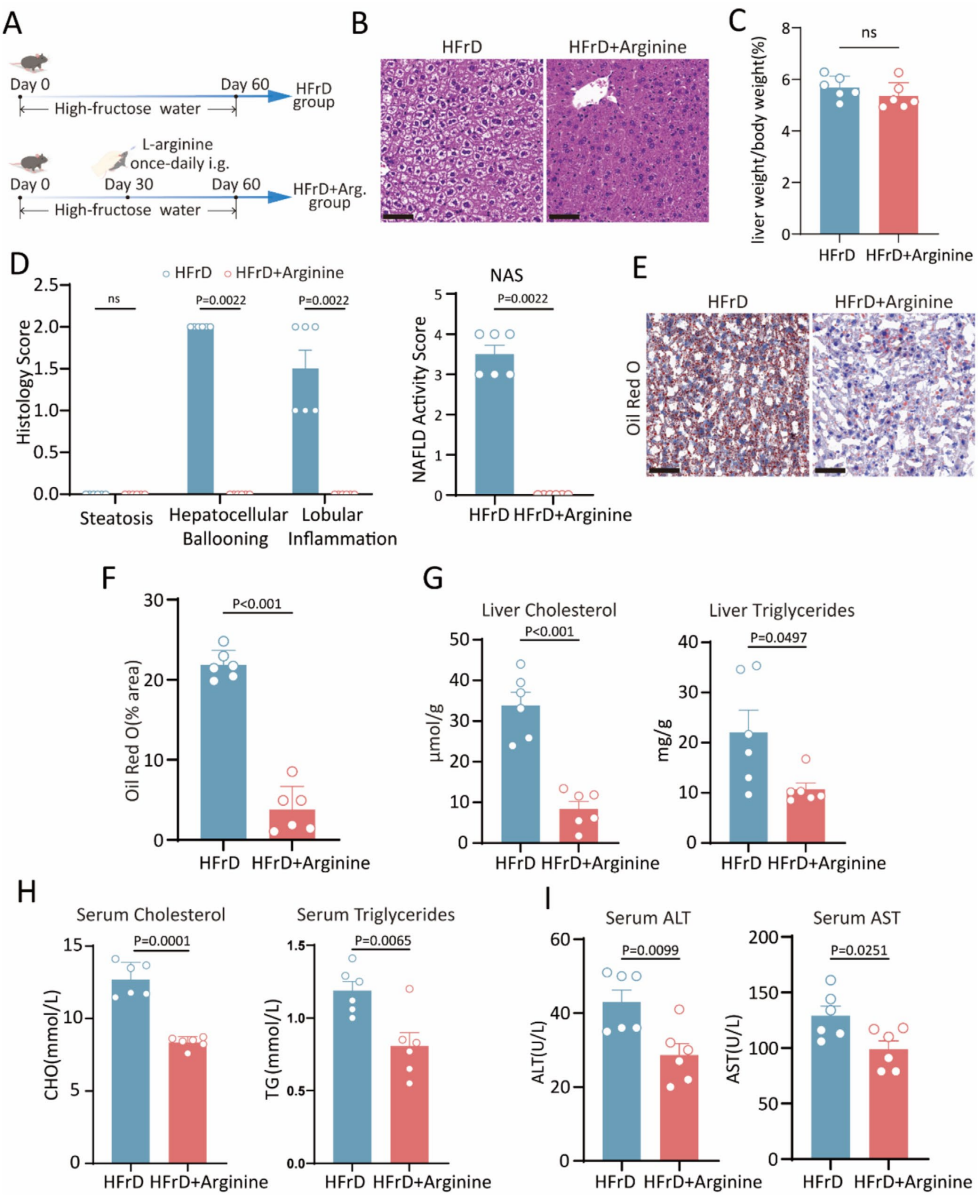
L‐arginine exhibited protective function against NAFLD in high fructose mice. (A) Schematic of arginine administration in HFrD mice. (B, D) H&E staining and quantitative analysis of liver sections. Scale bar, 60 μm. (C) Liver/body weight. (E, F) Representation images (E) and statistical analysis (F) of Oil Red O staining. Scale bar, 60 μm. (G, H) Triglycerides and cholesterol levels detection in liver (G) and serum (H). (I) Serum ALT and AST assay. Data represent mean ± SEM, compared to HFrD control mice, using unpaired *t*‐test. *n* = 6 mice per group for (A–I).

Logically, we are set to investigate whether arginine provides protection against high fructose‐induced MASLD. To this end, the HFrD mice were subjected to 4 weeks exogenous supplementation of L‐arginine (Figure 5A), and the MASLD‐related indicators were evaluated. The H&E staining results of liver sections demonstrated that while steatosis showed no significant difference, ballooning degeneration and lobular inflammation were markedly reduced in arginine‐treated HFrD mice, leading to a significant reduction in the NAS score (Figure 5B,D). The liver‐to‐body weight ratio showed no statistically significant difference between the HFrD group and the arginine‐supplemented HFrD group (Figure 5C). The number of lipid droplets in the liver of arginine‐treated HFrD mice was also significantly reduced compared to HFrD control mice, as confirmed by Oil Red O staining experiments (Figure 5E,F). The levels of triglycerides and cholesterol in both serum and liver were significantly lowered in HFrD mice that received arginine supplementation (Figure 5G,H). Consistently, arginine supplementation improved liver function in mice, as indicated by lower serum ALT and AST levels compared to those in the HFrD control mice (Figure 5I). In brief, arginine significantly alleviates high‐fructose induced MASLD. Combining the aforementioned results, which show a strong positive correlation between *L. murinus* and arginine, suggests that *L. murinus* exerts a protective effect against fructose‐induced MASLD through arginine.

## 4 Discussion

This study confirmed that a 3‐month high‐fructose drinking regimen results in pronounced MASLD. Both fructose‐supplemented drinking water and fructose‐enriched diet murine models are widely recognized and utilized in fructose‐related research (Richard et al. 2022; Zhang et al. 2023; Zhou et al. 2023; Fowle‐Grider et al. 2024). Early studies revealed that fructose, compared to glucose, is primarily metabolized in the liver and stimulates hepatic de novo lipogenesis (DNL) (Jensen et al. 2018; Geidl‐Flueck et al. 2021). Subsequently, a growing body of research has investigated fructose metabolism in vivo. Recent studies demonstrated that the small intestine also serves as a major site for fructose metabolism and clearance. Excessive fructose intake exceeding intestinal metabolic capacity leads to fructose spillover to the liver, thereby exacerbating hepatic DNL (Jang et al. 2018). Furthermore, research indicated that chronic overconsumption of fructose induces intestinal barrier impairment and endotoxemia. Circulating endotoxins bind to Toll‐like receptor 4 (TLR4), activating hepatic macrophages to produce tumor necrosis factor (TNF), which subsequently promotes DNL in hepatocytes (Todoric et al. 2020). Therefore, elucidating the complex mechanisms underlying fructose‐induced hepatic DNL is critical for developing targeted interventions against metabolic disorders.

Certain species of *Lactobacillus* have been widely reported to offer protective effects against the progression of high‐fat diet‐induced MASLD (Vallianou et al. 2021; Yu et al. 2021; Michels et al. 2022). Emerging evidence indicated that gut microbiota‐mediated conversion of dietary fructose to acetate fuels lipogenic acetyl‐CoA pools, exacerbating hepatic DNL. Microbiota depletion effectively suppressed fructose‐driven conversion of hepatic acetyl‐CoA and fatty acids (Zhao et al. 2020). However, the specific microbial taxa orchestrating HFrD‐induced pathological progression remain elusive. A translational human‐murine study identified reduced abundance of *Akkermansia* and *L. murinus* in patients with nonalcoholic fatty liver disease (NAFLD) compared to healthy controls (Lee et al. 2021). Mechanistic investigations in murine models demonstrated that supplementation with *L. acidophilus* , *L. fermentum* , and *L. plantarum* significantly attenuated high‐fat diet‐induced hepatic steatosis and cholesterol deposition (Lee et al. 2021). These data provided preclinical evidence that targeted modulation of *Lactobacillus* species may ameliorate NAFLD progression through microbiota‐dependent metabolic regulation.

To navigate the complexity and variability of the gut microbiota, we constructed two distinct mouse models—high‐fructose drinking and high‐fructose diet—to ensure the robustness of our findings. Two fructose feeding regimens induced model‐specific alterations in certain microbial taxa. These differences likely reflect the physiological distinctions between the two delivery methods, including the rate of fructose absorption, the duration of intestinal exposure, and interactions with dietary components. High‐fructose drinking water probably leads to faster and more concentrated fructose uptake in the proximal gut, thereby exerting a broader impact on the upper intestinal microbiota. In contrast, fructose from a solid diet is released more gradually and may interact with dietary fiber throughout the intestinal tract, imposing sustained selective pressure on bacterial groups. Our analysis consistently identified *L. murinus* as a critical gut microbial species influenced by prolonged fructose exposure across both administration paradigms. This reproducible depletion underscores that *L. murinus* is a central and reliable responder to fructose‐induced metabolic stress, enabling us to distinguish this key finding from model‐specific secondary effects. Therefore, we posit that the depletion of *L. murinus* represents a key microbial event in the pathogenesis of fructose‐induced MASLD. Furthermore, we confirmed that the administration of *L. murinus* significantly alleviates MASLD symptoms, establishing it as an unreported potential probiotic, the mechanisms of which merit further investigation.

The ‘gut‐liver axis’ describes the reciprocal interaction between the gut, microbial community, and the liver, which is important for understanding the development and progression of MASLD. The gut microbiota is essential for preserving the homeostasis of the ‘gut‐liver axis’ (Albillos et al. 2020; Tilg et al. 2022). Growing evidence suggests that metabolites derived from the microbiota, such as trimethylamine, secondary bile acids, short‐chain fatty acids, and ethanol, may exert effects on the pathogenesis of MASLD (Chen et al. 2016; Koh et al. 2016; Jiao et al. 2018). Our results revealed that arginine emerged as the sole co‐altered metabolite in both serum and feces of mice subjected to a long‐term fructose diet, and its levels were significantly positively associated with *L. murinus* abundance. Exogenous supplementation of *L. murinus* restored the host's arginine level and significantly improved MASLD‐related symptoms in fructose‐fed mice. Arginine acts as a critical substrate for multiple metabolic pathways, exerting profound regulatory effects on regeneration processes and immune responses (Martí i Líndez and Reith 2021, Starikova et al. 2023). Beyond its role in protein synthesis, arginine serves as a critical substrate for two competing metabolic pathways. It is metabolized by arginases to produce L‐ornithine, a precursor for polyamine synthesis, where polyamines play crucial roles in driving cellular proliferation and tissue repair (S. Clemente et al. 2020; Starikova et al. 2023). Alternatively, through NOS‐mediated metabolism, arginine is converted to nitric oxide (NO), which functions as an essential intracellular signaling molecule for maintaining vascular homeostasis and modulating immune cell function (Zhou and Zhu 2009; Mammedova et al. 2021). In the pathogenesis of MASLD, a characteristic metabolic shift occurs where elevated arginase activity diverts arginine toward polyamine synthesis, creating a functional arginine deficiency for NOS (Mammedova et al. 2021). This imbalance establishes a pathogenic cycle wherein reduced NO bioavailability exacerbates metabolic dysfunction, while simultaneously altered polyamine metabolism contributes to immunosuppressive microenvironment formation (Zhu et al. 2025). Therapeutic L‐arginine administration thus acts as a metabolic reservoir that concurrently restores NO‐dependent signaling for vascular function while supporting polyamine‐dependent processes for tissue repair and immune regulation (Starikova et al. 2023). The efficacy of arginine supplementation likely resides in recalibrating metabolic flux between these competing pathways rather than exclusively activating either one (Starikova et al. 2023). It is noteworthy that in our experimental model, L‐arginine supplementation significantly ameliorated hepatocellular ballooning and lobular inflammation without a concomitant reduction in steatosis scores. This differential therapeutic profile can be understood within the specific pathological context of our study. The 2‐month high‐fructose feeding protocol primarily induced the earlier, more rapidly manifesting hallmarks of MASLD—inflammatory injury and ballooning degeneration. Within this timeframe, the diet did not induce macrovesicular steatosis to a degree that exceeded the minimal histological scoring threshold, resulting in uniformly low steatosis scores across all experimental groups. Consequently, while our interventions demonstrably mitigated the dominant inflammatory pathology at this disease stage, the assessment of their impact on steatosis was inherently limited by its low baseline severity. Given the pivotal role of intracellular arginine metabolism in diverse pathological conditions—spanning immune disorders, cardiovascular diseases, and metabolic disorders—the modulation of arginine‐dependent pathways represents a promising therapeutic strategy. Our study identifies a novel association between arginine depletion and fructose‐induced MASLD, expanding the current understanding of arginine metabolism in metabolic diseases and highlighting the imperative to elucidate the precise mechanisms through which arginine modulates hepatic metabolism, which holds substantial translational potential for developing targeted therapeutic strategies.

Our findings identify a significant association between *L. murinus* depletion and altered arginine metabolism in the pathogenesis of high‐fructose‐induced MASLD. While the direct arginine‐synthesizing capability of *L. murinus* requires further investigation, our results can be contextualized by a recent groundbreaking discovery that *L. murinus* produces small RNAs capable of directly regulating host polyamine metabolism, for which arginine is a crucial precursor (Fan et al. 2024). This provides a compelling mechanistic precedent for our observed ‘ *L. murinus* ‐arginine’ axis. We postulate that the loss of *L. murinus* under high‐fructose feeding may disrupt this RNA‐mediated communication, leading to dysregulated polyamine metabolism and subsequent liver pathology.

Collectively, our results demonstrate that *L. murinus* plays a crucial role in counteracting fructose‐induced MASLD through mechanisms involving the restoration of host‐microbe arginine metabolism, thereby nominating this axis as a promising therapeutic target. While our findings highlight the potential of *L. murinus* and arginine as candidate therapeutic agents, their translational application requires careful consideration of several practical barriers. These include establishing the strain‐specificity of the observed benefits, overcoming challenges in the formulation, viability, and targeted delivery of live *L. murinus* , and determining the optimal dosing and long‐term safety profile of arginine supplementation in patients with metabolic liver disease. Future studies are therefore essential to validate these approaches in more complex models and human cohorts, and to explore potential synergistic effects before their clinical potential can be fully realized.

## Author Contributions

**Xinglin Mo:** formal analysis (equal), investigation (equal), methodology (equal), writing – original draft (equal). **Guilin Zhao:** data curation (equal), formal analysis (equal). **Lanlan Liu:** data curation (equal), investigation (equal). **Lan Zhen:** investigation (equal). **Qing Huang:** methodology (equal). **Yue Wang:** investigation (equal). **Xiaopan Yang:** software (equal), supervision (equal). **Linfei Huang:** investigation (equal). **Luming Wan:** project administration (equal), supervision (equal). **Congwen Wei:** conceptualization (equal), funding acquisition (equal), supervision (equal). **Ruzhou Zhao:** conceptualization (equal), funding acquisition (equal), writing – review and editing (equal). **Jie Hu:** funding acquisition (equal), supervision (equal). **Yong Li:** supervision (equal), writing – review and editing (equal). **Jing Yuan:** conceptualization (equal), supervision (equal), writing – review and editing (equal). **Chenke Ma:** supervision (equal), writing – original draft (equal), writing – review and editing (equal). **Feixiang Wu:** conceptualization (equal), project administration (equal), supervision (equal), writing – review and editing (equal).

## Funding

This work was supported by the National Natural Science Foundation of China (32300573), the National Natural Science Foundation of China (NSFC) (82360537), Key Laboratory of Early Prevention and Treatment for Regional High Frequency Tumor (Guangxi Medical University), Ministry of Education/Guangxi Key Laboratory of Early Prevention and Treatment for Regional High Frequency Tumor (GKE‐ZZ202309), PLA General Hospital Youth Independent Innovation Science Fund—Growth project (22QNCZ042).

## Ethics Statement

The studies involving animal experiments were conducted at the AMMS Animal Center and were approved by the Ethics Committee of Beijing Institute of Biotechnology (IACUC‐DWZX‐2023‐P009).

## Conflicts of Interest

The authors declare no conflicts of interest.

## Supporting information


**Data S1:** fsn371502‐sup‐0001‐SupinfoS1.xlsx.

## Data Availability

All untargeted metabolomic data and targeted metabolomic data used in this publication have been deposited to the EMBL‐EBI MetaboLights database with the identifier MTBLS12248 (colonic contents metabolomics) and MTBLS12249 (serum metabolomics). The complete data set can be accessed at https://www.ebi.ac.uk/metabolights/MTBLS12248 and https://www.ebi.ac.uk/metabolights/MTBLS12249. The raw reads files for each sample of 16S rDNA sequencing have been uploaded to the NCBI Sequence Read Archive (SRA) under the project number PRJNA1226916.
